# Establishment and associated factors of health records among young Chinese migrants

**DOI:** 10.1186/s12199-021-00961-1

**Published:** 2021-03-24

**Authors:** Hong Shi, Xiumin Zhang, Xiangrong Li, Zheng Ren, Hanfang Zhao, Minfu He, Xinwen Fan, Xia Guo, Shuang Zha, Shuyin Qiao, Yuyu Li, Yajiao Pu, Hongjian Liu

**Affiliations:** 1 Department of Social Medicine and Health Management, School of Public Health, Jilin University, Changchun, 130021 China; 2 Department of Epidemiology and Biostatistics, School of Public Health, Jilin University, Changchun, 130021 China

**Keywords:** Young migrants, Health records, Associated factors

## Abstract

**Background:**

Most Chinese migrants have been faced with obstacles to getting access to local public health services. Young migrants among internal migrants make a major contribution to the economy. However, the establishment of their health records has been ignored. This research was aimed at exploring the status and associated factors of the establishment of health records among young Chinese migrants.

**Methods:**

Data were obtained from the 2017 China Migrants Dynamic Survey (CMDS). Chi-square test and binary logistic regression were performed to investigate the associated factors of the establishment of health records among young Chinese migrants.

**Results:**

Approximately 30.2% of young migrants had their health records established in inflow communities. Urban residence, medical insurance (insured), and lower average monthly household income were attributed to the establishment of more health records. Young migrants who were in northeast China and across provinces and immigrated for working or engaging in trade were less likely to have health records established. Young migrants who participated in social activities and public affairs activities and took type of people in touch as natives in the inflow area showed a higher possibility of establishing health records. Meanwhile, receiving health education and hearing about national basic public health services (BPHSs) were beneficial for establishing the health records of more young migrants.

**Conclusion:**

This study showed that the health records of young migrants had a relatively low establishment rate. Sociodemographic and migration characteristics, health status, public health services, and social integration factors were all related to the health record establishment of young Chinese migrants.

## Background

Changes have taken place in urban and rural population structures due to the current construction of new urbanization. Migration is the core issue of the recent urbanization process of China [1]. The 2018 Report on China’s Migrant Population Development revealed that the number of migrants in China reached up to 244 million in 2017 [2]. Young migrants among internal migrants make a major contribution to the economy and have a direct or indirect impact on the development of politics, economy, culture, and other aspects in China [3]. Concurrently, research has shown that younger migrants are most likely to experience a decline in health after migration [4]. Furthermore, they are much less likely to be aware of their conditions, suffer from unnoticed high health risks leading to their vulnerability to long-term health problems [5], and find it impossible to seek medical attention by themselves [6]. A worrying finding is that migration characteristics and inattention to health result in the vulnerability of young migrants to ill health. Given a large number of Chinese migrants, it is urgent to address the health problems brought by migration, which poses particular demands on the basic public health service system.

“Equalization of Essential Public Health Services” is a rare national program that covers the prevention and control of non-communicable diseases (NCDs) for internal migrants [7]. The latest basic public health services (BPHS) package includes 14 categories which involve establishing original items like health records and health education, adding health supervision assistance, and promoting free contraceptives and health literacy [8]. As the main item of the equalization of BPHSs, health records include basic personal information, health examination information, health management of key populations and other medical and health service records. Specifically, basic personal information includes name, gender, education level, marital status, basic health information, etc. Health examination information includes general health examination, lifestyle, health status, etc. Health management of key populations mainly refers to the health management records of children aged 0–6, pregnant women, the elderly and individuals with chronic diseases, etc. Other medical and health service records include the records of receive and transfer. The establishment of health records is funded by the state and provided to residents free of charge. The residents’ health records are coded uniformly, and managed by community health centers. Health records are the basis for implementing other health services, play a significant role in providing sufficient access to public health services, work as management tools assisting medical staff in storing, accessing and managing the personal health information of residents so as to make decisions on their health status [9], effectively control health risk factors and enable patients and health providers to make right decisions on health management or treatment [10].

In response to the right and health protection of migrants, “Healthy China 2030” explicitly proposes to promote the equalization of BPHSs to gradually narrow the differences in basic health services and the inequality of health between populations [11]. “13th Five-Year Plan for Health and Wellness” has made clear demands to facilitate the better management of migrants and promote the equity of BPHSs [12]. Despite the great emphasis placed in the national policy on the equity between migrants and residents, most migrants are excluded from local public health services in the cities where they live. Existing literature has indicated that people who have been working or living outside for a long time encounter barriers when getting access to BPHSs due to their weak health awareness and failure to establish health records [13]. That is to say, migrants cannot enjoy basic health services like local residents because of migration status.

As highlighted above, it is of importance to promote the above-mentioned national program and establish the health records of more young migrants. However, data about the situation of young migrants are minimal because most of the research has focused on the migrants who are ordinary and elderly and suffer from chronic diseases [9, 14, 15]. Moreover, a number of previous studies emphasized the socio-economic and demographic characteristics affecting health records establishment [16, 17]. Considering the potential impact of more comprehensive migration characteristics and social integration factors, this analysis was aimed at exploring the status and associated factors of the establishment of health records among young Chinese migrants by using national survey data in 2017, which can help to identify the barriers to the health record establishment of young migrants and play a crucial role in providing robust evidence and recommendations for targeted interventions to establish more health records.

## Methods

### Data source and research sample

Data were retrieved from the 2017 China Migrants Dynamic Survey (CMDS), a cross-sectional and nationally representative survey organized by the National Health Commission of the People’s Republic of China [18]. A total of 169,989 subjects were recruited, who were at the age of 15 and above, lacked a local Hukou (rural or urban residence) and had dwelled in their current location for no less than 1 month.

The subjects of this study are young migrants. The World Health Organization limits the age of young people to 44 [19]. Young migrants in this study refer to the migrants aged between 15 and 44. With regard to the idea of this study, the management and service specification of residents’ health records states that subjects are those who have lived in their current residence for over 6 months with a local Hukou. Young migrants who had settled in their current residence for less than half a year were excluded. As a result, a final sample of 115,730 was obtained.

### Variables

#### Dependent variables

The establishment status of the health records of young migrants was measured by the survey question “Have you established your health records in your community?” The establishment of health records was coded as “yes=1” if the reply was “Yes, established”, and “no=0” if the answer was “No, I’ve never heard of it”, “No, but I’ve heard it” or “I don't know about it” [20, 21].

#### Independent variables

Sociodemographic characteristics included gender (male or female), age (15–29 or 30–44), ethnicity (Han or minority), marital status (single (unmarried, widowed or divorced), or married (first or non-first marriage)), Hukou status, education level (primary school or below or middle/high school/college degree or above), medical insurance (uninsured/insured), and average monthly household income (below 3000/3001–5000/above 5001 yuan).

Migration characteristics were measured through five migrant-related variables, namely sample area (east/central/west/northeast), extent (inter-provincial/intra-provincial/inter-country within the city) and duration of migration (≤ 1 year/2–4 years/5–9 years/≥ 10 years), reasons for migration (working or engaging in trade/following the migration of family members/marriage-related/else) as well as settlement willingness (no/yes).

Health status and public health services factors included self-rated health (good/neutral/poor), hypertension or type 2 diabetes (no/yes), distance of medical facilities (≤15 min/> 15 min), hearing about national BPHSs (no/yes) and health education (no/yes). The status of health education was measured by the survey question “Did you receive health education in your community last year?” whose answers have the following nine items: Occupational disease prevention, sexually transmitted disease (STD)/acquired immune deficiency syndrome (AIDS) prevention, reproductive health and contraception, tuberculosis prevention, smoking control, mental health, chronic disease prevention, maternal and child health care as well as self-help in public emergencies. Health education was coded as “no=0” if none of the above items were selected and otherwise “yes=1.”

According to existing studies and available data [22–24], four variables were constructed to measure the social integration of migrants, including the participation in social activities and public affairs activities, willingness to integrate and type of people in touch. Four questions that were selected to measure the social integration of migrants are as follows. (1) “Have you ever participated in the activities of the following organizations in the inflow area?” whose answers have the following six items: Union, Volunteer’s Association, Alumni Association, Townsmen Association, Hometown Chamber of Commerce, and others. Participation in social activities was coded as “no=0” if none of the above activities were attended and otherwise “yes=1.” (2) “Have you ever had any of the following behaviors?” whose answers have the following five items: advise or supervise community or village management, provide government departments with policy recommendations, and participate in discussions on state affairs or social events online, donation, blood donation, and volunteer activities as well as party branch and league activities. The participation in public affairs activities was coded as “no=0” if none of the above behaviors appeared and otherwise “yes=1.” (3) “Do you agree that you are willing to be a part of local residents.” The willingness to integrate was coded as “no=0” if the response was “totally disagree” or “disagree” and “yes=1” if the response was “agree” or “totally agree.” (4) Type of people in touch was measured by the survey question “In your spare time, who do you associate with most in the inflow area,” and coded as “people from the same hometown=1,” “natives in the inflow area=2,” “immigrants from places other than hometown=3,” and “little contact with others=4.”

### Statistical analysis

Statistical analyses were carried out by using Statistical Product and Service Solutions (SPSS) 24.0 (IBM Corp, Armonk, NY, USA). Demographic information and the establishment status of health records were described using descriptive statistical analysis. The difference in the health record establishment of young migrants with various characteristics was analyzed by performing a chi-square test. Multivariate analysis was tested by binary logistic regression. The associated factors of the establishment of health records among young migrants in China were further investigated by performing binary logistic regression and using the odd ratio (OR) and 95% confidence intervals (CIs). A two-side *p* value less than 0.05 was considered as statistically significant. Before conducting binary logistic regression, independent variables were tested for co-linearity according to tolerance values and the VIF (variance inflation factor). The criteria values for tolerance and VIF (≤ 0.10 and ≥ 10, respectively) to identify co-linearity [25].

Since the establishment of health records is classified as either “yes” or “no,” a binary logistic model was used to fit the data and explore the associated factors of the establishment of health records among young Chinese migrants. The formula of binary logistic regression is as follows:

$$ Logit(p)=\mathit{\ln}\left[\frac{p}{1-p}\right]=\alpha +{\beta}_1{X}_1+{\beta}_2{X}_2+\cdots +{\beta}_m{X}_m $$

where *p* denotes the probability of establishing health records, *α* is a constant term, and the fitting regression coefficients are expressed as *β*_*j*_ *(j=1, 2, …, m)*, the potential explanatory variables are expressed as *X*_*j*_ *(j=1, 2, …, m).* The OR indicator is able to be directly calculated in logistic regression by *OR = e*^*β*^.

## Results

### Characteristics of young migrants

50.8% were females among 115,730 young migrants who were aged 32.00 on average, with a standard deviation of 6.50. Most participants were rural (82.8%) and married (79.8%) with a middle school diploma (43.3%). Most young migrants bought medical insurance (91.7%), among whom about 58.2% had an average monthly household income of above 5001 yuan. As for migration characteristics, more young internal migrants (41.7%) were in eastern regions, and most of the participants were inter-provincial young migrants (47.8%), with the duration of migration 2–4 years (33.3%). The sample population of this study immigrated for working or engaging in trade (84.2%), with settlement willingness (84.2%). With regard to health status and PHS characteristics, the majority of young migrants had good self-rated health (87.0%), about 2.0% had hypertension or type 2 diabetes, the distance of medical facilities was ≤ 15 min (84.2%), 61.8% had heard about national BPHSs, and most received health education (75.1%). Regarding social integration, 49.0% of young migrants participated in social activities, 45.2% participated in public affairs activities, 93.7% were willing to integrate and type of people in touch was natives in the inflow area (34.5%).

### Establishment status of health records

The results indicated that about 30.2% of young migrants established their health records in their local communities. Establishment rate of health records among young migrants in the east, central, west, and northeast areas were 26.0%, 41.3%, 30.9%, and 24.7%, respectively.

### Factors associated with the establishment status of health records

Univariate analysis indicated that gender, age, marital status, Hukou status, education level, medical insurance, and average monthly household income were significantly associated with health record establishment (Table 1).

**Table 1:** Sociodemographic characteristics and the establishment of health records (*N* = 115,730)

Variables	Total ***N*** (%)	Establishment of health records		***χ***^**2**^	***p***
		Yes	No		
Gender				97.523	< 0.001
Male	56,921 (49.2)	16,429 (28.9)	40,492 (71.1)		
Female	58,809 (50.8)	18,542 (31.5)	40,267 (68.5)		
Age				18.004	< 0.001
15–29	44,206 (38.2)	13,036 (29.5)	31,170 (70.5)		
30–44	71,524 (61.8)	21,935 (30.7)	49,589 (69.3)		
Ethnicity				1.477	0.224
Han	104,512 (90.3)	31,525 (30.2)	72,987 (69.8)		
Minority	11,218 (9.7)	3446 (30.7)	7772 (69.3)		
Marital status				251.598	< 0.001
Married	92,385 (79.8)	28,911 (31.3)	63,474 (68.7)		
Single	23,345 (20.2)	6060 (26.0)	17,285 (74.0)		
Hukou status				60.538	< 0.001
Rural	95,801 (82.8)	28,490 (29.7)	67,311 (70.3)		
Urban	19,929 (17.2)	6481 (32.5)	13,448 (67.5)		
Education level				150.702	< 0.001
Primary school or below	12,066 (10.4)	3265 (27.1)	8801 (72.9)		
Middle school	50,100 (43.3)	14,658 (29.3)	35,442 (70.7)		
High school	27,998 (24.2)	8782 (31.4)	19,216 (68.6)		
College degree or above	25,566 (22.1)	8266 (32.3)	17,300 (67.7)		
Medical insurance				319.981	<0.001
Insured	10,6078 (91.7)	32,827 (30.9)	73,251(69.1)		
Uninsured	9652 (8.3)	2144 (22.2)	7508 (77.8)		
Average monthly household income				11.666	0.003
≤ 3000	14,325 (12.4)	4173 (29.1)	10,152 (70.9)		
3001–5000	34,054 (29.4)	10,452 (30.7)	23,602 (69.3)		
≥ 5001	67,351 (58.2)	20,346 (30.2)	47,005 (69.8)		

Univariate analysis indicated that sample area, extent, and duration of migration, reasons for migration and settlement willingness were significantly related to health record establishment (Table 2).

**Table 2:** Migration characteristics and the establishment of health records (*N* = 115,730)

Variables	Total ***N*** (%)	Establishment of health records		***χ***^**2**^	***p***
		Yes	No		
Sample area				1699.815	< 0.001
East	48,289 (41.7)	12,548 (26.0)	35,741 (74.0)		
Central	20,210 (17.5)	8337 (41.3)	11,873 (58.7)		
West	39,221 (33.9)	12,106 (30.9)	27,115 (69.1)		
Northeast	8010 (6.9)	1980 (24.7)	6030 (75.3)		
Extent of migration				1012.122	< 0.001
Inter-provincial	55,282 (47.8)	14,225 (25.7)	41,057 (74.3)		
Intra-provincial	39,377 (34.0)	13,438 (34.1)	25,939 (65.9)		
Inter-country within the city	21,071 (18.2)	7308 (34.7)	13,763 (65.3)		
Duration of migration				93.896	< 0.001
≤ 1	18,536 (16.0)	5196 (28.0)	13,340 (72.0)		
2–4	38,532 (33.3)	11,925 (30.9)	26,607 (69.1)		
5–9	34,861 (30.1)	10,957 (31.4)	23,904 (68.6)		
≥10	23,801 (20.6)	6893 (29.0)	16,908 (71.0)		
Reasons of migration				207.260	< 0.001
Working or engaging in trade	97,395 (84.2)	28,652 (29.4)	68,743 (70.6)		
Following the migration of family members	10,415 (9.0)	3526 (33.9)	6889 (66.1)		
Marriage-related	3660 (3.2)	1374 (37.5)	2286 (62.5)		
Else	4260 (3.7)	1419 (33.3)	2841 (66.7)		
Settlement willingness				330.920	< 0.001
Yes	97,473 (84.2)	30,490 (31.3)	66,983 (68.7)		
No	18,257 (15.8)	4481 (24.5)	13,776 (75.5)		

Univariate analysis indicated that self-rated health, distance of medical facilities, health education, and hearing about national BPHSs were significantly associated with health record establishment (Table 3).

**Table 3:** Health status and public health services factors and the establishment of health records (*N* = 115,730)

Variables	Total ***N*** (%)	Establishment of health records		***χ***^**2**^	***p***
		Yes	No		
Self-rated health				114.668	< 0.001
Good	100,693 (87.0)	30,985 (30.8)	69,708 (69.2)		
Neutral	13,834 (12.0)	3688 (26.7)	10,146 (73.3)		
Poor	1203 (1.0)	298 (24.8)	905 (75.2)		
Hypertension or type 2 diabetes				0.655	0.418
Yes	2339 (2.0)	689 (29.5)	1650 (70.5)		
No	113,391 (98.0)	34,282 (30.2)	79,109 (69.8)		
Distance of medical facilities				148.509	< 0.001
≤ 15 min	97,403 (84.2)	30,128 (30.9)	67,275 (69.1)		
>15 min	18,327 (15.8)	4843 (26.4)	13,484 (73.6)		
Health education				7,195.044	< 0.001
Yes	86,967 (75.1)	32,006 (36.8)	54,961 (63.2)		
No	28,763 (24.9)	2965 (10.3)	25,798 (89.7)		
Hearing about national BPHSs				20,561.108	< 0.001
Yes	71,479 (61.8)	32,485 (45.4)	38,994 (54.6)		
No	44,251 (38.2)	2486 (5.6)	41,765 (94.4)		

Univariate analysis indicated that participation in social activities and public affairs activities, willingness to integrate and type of people in touch were significantly related to health record establishment (Table 4).

**Table 4:** Social integration characteristics and the establishment of health records (*N* = 115,730)

Variables	Total ***N*** (%)	Establishment of health records		***χ***^**2**^	***p***
		Yes	No		
Participation in social activities				1859.835	< 0.001
Yes	56,653 (49.0)	20,487 (36.2)	36,166 (63.8)		
No	59,077 (51.0)	14,484 (24.5)	44,593(75.5)		
Participation in public affairs activities				1405.301	< 0.001
Yes	52,289 (45.2)	18,715 (35.8)	33,574 (64.2)		
No	63,441 (54.8)	16,256 (25.6)	47,185 (74.4)		
Willingness to integrate				191.433	< 0.001
Yes	108,495 (93.7)	33,308 (30.7)	75,187 (69.3)		
No	7235 (6.3)	1663 (23.0)	5572 (77.0)		
Type of people in touch				547.330	< 0.001
People from the same hometown	39,097 (33.8)	11,266 (28.8)	27,831 (71.2)		
Natives in the inflow area	39,975 (34.5)	13,774 (34.5)	26,201 (65.5)		
Immigrants from places other than hometown	13,621 (11.8)	3713 (27.3)	9908 (72.7)		
Little contact with others	23,037 (19.9)	6218 (27.0)	16,819 (73.0)		

A co-linearity analysis showed a VIF < 10 and no co-linearity among the independent variables (Table 5). Table 6 presents the binary logistic regression results of factors that were related to the establishment of health records among young migrants and also identified as variables in the multivariate analysis if observed to be correlated with the health record establishment of young migrants in univariate analysis. The binary logistic regression indicated that young migrants were more inclined to have established their health records if female; married and insured; and having urban residence; a primary school diploma or below; and an average monthly household income of less than 3000 yuan. Those who were northeast and inter-province young migrants, had duration of migration ≤ 1 year and immigrated for working or engaging in trade without settlement willingness were less likely to have established health records. Good self-rated health, distance of medical facilities ≤ 15 min, health education and hearing about national BPHSs were positive factors. Meanwhile, young migrants who participated in social activities and public affairs activities and took type of people in touch as natives in the inflow area had a higher likelihood of having established health records.

**Table 5:** Co-linearity analysis of factors that associated with health records establishment of young Chinese migrants

Variables	Collinear statistics		Variables	Collinear statistics	
	Tolerance	VIF		Tolerance	VIF
Gender	0.928	1.078	Reasons of migration	0.928	1.077
Age	0.731	1.368	Settlement willingness	0.946	1.057
Marital status	0.730	1.370	Self-rated health	0.975	1.025
Hukou status	0.837	1.195	Distance of medical facilities	0.989	1.011
Education level	0.734	1.362	Health education	0.856	1.168
Medical insurance	0.967	1.034	Hearing about national BPHSs	0.873	1.145
Average monthly household income	0.819	1.221	Participation in social activities	0.828	1.208
Sample area	0.878	1.140	Participation in public affairs activities	0.857	1.167
Extent of migration	0.895	1.117	Willingness to integrate	0.968	1.033
Duration of migration	0.894	1.118	Type of people in touch	0.961	1.041

*VIF* Variance inflation factor

**Table 6:** Binary logistic regression analysis of factors associated with health records establishment of young Chinese migrants (*N* = 115,730)

Variables	***B***	***p***	OR	95% CI	
				Lower	Upper
Gender (Ref = male)	0.049	0.001	1.050	1.019	1.082
Marital status (Ref = single)	0.194	< 0.001	1.214	1.166	1.263
Hukou status (Ref = rural)	0.086	< 0.001	1.090	1.046	1.135
Education level (Ref = primary school or below)					
Middle school	− 0.095	< 0.001	0.910	0.863	0.958
High school	− 0.156	< 0.001	0.856	0.809	0.906
College degree or above	− 0.196	< 0.001	0.822	0.774	0.874
Medical insurance (Ref = uninsured)	0.163	< 0.001	1.177	1.112	1.246
Average monthly household income ( Ref = ≤ 3000)					
3001–5000	− 0.036	0.153	0.964	0.918	1.014
≥5001	− 0.116	< 0.001	0.891	0.848	0.935
Sample area (Ref = northeast)					
East	0.046	0.160	1.047	0.982	1.116
Central	0.477	< 0.001	1.611	1.507	1.722
West	0.072	0.025	1.075	1.009	1.145
Extent of migration (Ref = inter-provincial)					
Intra-provincial	0.176	< 0.001	1.192	1.153	1.233
Inter-country within the city	0.099	< 0.001	1.104	1.058	1.151
Duration of migration (Ref = ≤ 1)					
2–4	0.096	< 0.001	1.100	1.053	1.150
5–9	0.109	< 0.001	1.115	1.066	1.167
≥ 10	0.048	0.055	1.050	0.999	1.103
Reasons of migration (Ref = working or engaging in trade)					
Following the migration of family members	0.206	< 0.001	1.229	1.168	1.293
Marriage-related	0.162	< 0.001	1.175	1.086	1.272
Else	0.149	< 0.001	1.161	1.077	1.251
Settlement willingness (Ref = no)	0.171	< 0.001	1.187	1.138	1.237
Self-rated health (Ref = good)					
Neutral	− 0.228	< 0.001	0.796	0.761	0.833
Poor	− 0.080	0.305	0.923	0.793	1.075
Distance of medical facilities (Ref = > 15 min)	0.114	< 0.001	1.121	1.077	1.167
Health education (Ref = no)	0.980	< 0.001	2.664	2.548	2.784
Hearing about national BPHSs (Ref = no)	2.382	< 0.001	10.824	10.359	11.311
Participation in social activities (Ref = no)	0.204	< 0.001	1.226	1.189	1.265
Participation in public affairs activities (Ref = no)	0.138	< 0.001	1.149	1.114	1.184
Type of people in touch (Ref = little contact with others)					
People from the same hometown	− 0.067	0.002	0.935	0.896	0.975
Natives in the inflow area	0.069	0.001	1.072	1.028	1.118
Immigrants from places other than hometown	− 0.031	0.270	0.970	0.918	1.024

## Discussion

The designated policy pointed out that health records should keep an establishment rate of above 75% [15]. Previous studies have shown that the health records of residents in urban and rural areas had an establishment rate of 80.6% [13], satisfying the policy requirement. However, this study found that 30.2% of young migrants had established health records in their local communities, which fell below the national recommendation. Furthermore, the health record establishment rate of young migrants was relatively lower than that of the general population. Undeniably, Chinese migrants and residents show inequalities in the utilization of BPHSs. Considering the importance of health status and the low establishment rate of health records among young migrants, it is necessary to make targeted interventions to help to establish more health records based on associated factors.

In line with previous research, young migrants with a non-agricultural Hukou had a high possibility of establishing health records, which may be because migrants with an agricultural Hukou are more marginalized in many ways [9]. Basic medical insurance is the major source of healthcare financing in China, including the Urban Employee Basic Medical Insurance, the New Rural Cooperative Medical Scheme, and the Urban Residents Basic Medical Insurance. The insurance policies, including the insurance premium and reimbursement coverage, are different to the various target populations [26]. The household registration system directly affected the participation and use of medical insurance [27]. Young migrants who were insured were found to have a higher possibility of establishing health records, which was consistent with prior studies [14]. Young migrants with medical insurance may tend to be more aware of their own health initiatively. Therefore, policy-makers should pay more attention to the living conditions and improvement of utilization level of public health services of young migrants in agricultural Hukou. It was observed that middle school or above education and higher household income had negative impact on the health record establishment of young migrants. This result is contrary to the result of univariate analysis. The possible reason is that education level and average monthly household income may be confounded by other factors in univariate analysis. For the high levels of employment benefits and medical benefits, those migrants who with higher education level would be less likely to go to community health center. Moreover, they might have more stressful work, and thus have less time to make use of public health services including the establishment of health records. Young migrants whose household income was higher would be more likely to visit doctors in hospitals rather than community health centers. They would be more likely to use high-quality health services, which would possibly lead to their neglect of health records [28]. In recent years, the Chinese government has taken several measures to promote equal access to basic public health services among residents and migrants. Therefore, the migrants with low socioeconomic status had positive impact on the health record establishment, which reflects the goal of equalization of basic public health services.

Migration characteristics substantially contributed to the health inequality among young migrants who tend to be vulnerable to poor health conditions and utilize insufficient BPHSs due to external environmental factors and internal cultural shock in the cities where they live. It was noticed that northeast and inter-province migrant status, duration of migration ≤ 1 year, working or engaging in the trade as the reason for migration and no settlement willingness had a negative impact on the health record establishment of young migrants, which was parallel with previous studies [14, 29]. It is likely that environments and conditions in different areas were diverse. Migrants were faced with challenges navigating a new and unfamiliar health system [30]. Young internal migrants in northeastern regions showed a lower establishment rate of health records than those in central and western China, which was attributed to the economic level and distribution of health resources among different regions. Meanwhile, it is possible that young migrants owning short-term residence and immigrating for working or engaging in trade had less personal time and health care awareness to comprehend local specific health and preventive information, and little motivation to establish health records. Based on the above findings, it is essential to optimize the allocation of corresponding health elements in northeastern regions to narrow regional gaps, and goal-oriented policies and ensuring mechanisms should be made to enhance fairness of financing so as to promote accessibility and equality. Community health centers should strengthen propaganda and guidance to heighten the understanding of local health policies and establish the health records of more young migrants.

Young migrants who received health education and heard about national BPHSs showed a higher likelihood of establishing health records. These results indicated that health education and hearing about national BPHSs had positive impact on the health record establishment of young migrants. Apart from helping to improve the health conditions of young migrants indirectly by identifying their health risk factors, keeping healthy habits and shaping individual behavior, health education assists young migrants in gaining a better understanding of the advantages of establishing health records, removes privacy concerns and social distrust, and advances the health literacy and health consciousness of young migrants gradually. However, only less than 50% of migrants with health education or who know about national BPHSs established health records. These results suggested that providing health education or providing information on national BPHSs was not enough to establish health record in young Chinese migrants. Therefore, it is essential to adopt effective methods to implement health education and promotion in the community [18, 31]. Specifically, materials about BPHSs should be posted in the publicity column of the community, to popularize the ways and functions of establishing health records, meanwhile, electronic information-based health education channels including the Internet and new media should be developed to improve the quality of health education. Strengthening health literacy and adequate public health knowledge of young migrants aims to significantly promote the establishment of health records. Moreover, other factors should also be considered to increase the proportion of young Chinese migrants who establish health record.

Previous studies reported that community activities were conducive to improving the accessibility of health services among internal migrants [32]. The results of this study showed that young migrants participating in social activities and public affairs activities were more inclined to establish health records than those without. In addition, natives in the inflow area as type of people in touch made significant positive contributions to health record establishment. High-integrated young migrants were more familiar with community health centers and acceptable to the health information of current residence. Health information becomes an enabling factor that promotes the utilization of public health services. The reasons for migration were parallel with previous studies, suggesting that internal migrants who had better social integration were more inclined to utilize public health services [29]. Young migrants who had social interaction with natives in the inflow area could get more functional and emotional social support [33] to develop self-identity and a sense of social belonging. Meanwhile, natives in the inflow area can help them to be informed of local preventive measures, inevitably affecting the establishment of health records among young migrants in inflow communities. Therefore, the community should notice the importance of more social cohesion among young migrants [34]. A variety of community activities should be organized to create an enthusiastic environment. More interventions should be implemented to encourage positive social adaptation and interaction between natives in the inflow area and young migrants.

## Limitations

The limitations of this study should be considered. First, cross-sectional data were used, preventing the interpretation of causal relationships. Second, subjective biases cannot be ruled out because of self-reported information on the establishment of health records and status. Thirdly, some variables considered in our study cannot be modified. There are no effective interventions to establish health records based on these variables. More modifiable factors should be further studied in future. Finally, the factors which were associated with the adoption of health records should be identified from the perspectives of health service providers and users. This study was based on the associated factors of health service users. More essential potential factors of health service providers should be further studied and confirmed in follow-up studies.

## Conclusion

This study showed that the health records of young migrants had a relatively low establishment rate. Sociodemographic and migration characteristics, health status, public health services, and social integration factors were all associated with health record establishment. Due to heterogeneity, policies and interventions should take into account different subgroups, especially vulnerable ones, strengthen the impact of health education on the popularization of health knowledge and the establishment of health records, and improve the health level of young migrants. The multifaceted association suggested that health administrations, communities, and young migrants need to make joint efforts of varying degrees to strengthen the equalization of BPHSs, and form external recourse supply and internal active participation to promote the establishment rate of health records.

## Data Availability

The datasets used and/or analyzed during the current study are available from the corresponding author on reasonable request.

## Abbreviations

NCDs: Non-communicable diseases
BPHS: Basic public health services
CMDS: China Migrants Dynamic Survey
STD: Sexually transmitted disease
AIDS: Acquired immune deficiency syndrome
